# Acute Pre-/Post-Treatment with 8th Day SOD-Like Supreme (a Free Radical Scavenging Health Product) Protects against Oxidant-Induced Injury in Cultured Cardiomyocytes and Hepatocytes In Vitro as Well as in Mouse Myocardium and Liver In Vivo

**DOI:** 10.3390/antiox6020028

**Published:** 2017-04-10

**Authors:** Pou Kuan Leong, Jihang Chen, Wing Man Chan, Hoi Yan Leung, Lincoln Chan, Kam Ming Ko

**Affiliations:** 1 Division of Life Science, The Hong Kong University of Science and Technology, Clear Water Bay, Kowloon, Hong Kong SAR, China; eriol@ust.hk (P.K.L.); cjh@ust.hk (J.C.); wingman@ust.hk (W.M.C.); hoiyan@ust.hk (H.Y.L.); 2 Health Science Group Holdings Limited, Hong Kong SAR, China; lincoln.chan@proddint.com

**Keywords:** antioxidant, free radical scavenger, oxidative stress, glutathione, myocardium, liver

## Abstract

8th Day superoxide dismutase (SOD)-Like Supreme (SOD-Like Supreme, a free radical scavenging health product) is an antioxidant-enriched fermentation preparation with free radical scavenging properties. In the present study, the cellular/tissue protective actions of SOD-Like Supreme against menadione toxicity in cultured H9c2 cardiomyocytes and in AML12 hepatocytes as well as oxidant-induced injury in the mouse myocardium and liver were investigated. SOD-Like Supreme was found to possess potent free radical scavenging activity in vitro as assessed by an oxygen radical absorbance capacity assay. Incubation with SOD-Like Supreme (0.5–3% (v/v)) was shown to protect against menadione-induced toxicity in H9c2 and AML12 cells, as evidenced by increases in cell viability. The ability of SOD-Like Supreme to protect against menadione cytotoxicity was associated with an elevation in the cellular reduced glutathione (GSH)/oxidized glutathione (GSSG) ratio in menadione-challenged cells. Consistent with the cell-based studies, pre-/post-treatment with SOD-Like Supreme (0.69 and 2.06 mL/kg, three intermittent doses per day for two consecutive days) was found to protect against isoproterenol-induced myocardial injury and carbon tetrachloride hepatotoxicity in mice. The cardio/hepatoprotection afforded by SOD-Like Supreme was also paralleled by increases in myocardial/hepatic mitochondrial GSH/GSSG ratios in the SOD-Like Supreme-treated/oxidant-challenged mice. In conclusion, incubation/treatment with SOD-Like Supreme was found to protect against oxidant-induced injury in vitro and in vivo, presumably by virtue of its free radical scavenging activity.

## 1. Introduction

The mitochondrial free radical theory of aging states that reactive oxygen species (ROS) arising from mitochondrial respiration are likely to cause damage to structural and functional components of mitochondria, favoring the pathogenesis of age-associated diseases and acceleration of the aging process [1,2]. In addition to mitochondrial respiration, ROS can also be generated by the cytochrome P450 (CYP)-catalyzed metabolism of xenobiotics in the liver [3]. To cope with these ROS, the body is equipped with antioxidant systems, which consist of exogenous free radical scavengers (such as, ascorbic acid and α-tocopherol), endogenous free radical scavengers (such as, reduced glutathione (GSH) and thioredoxin), as well as antioxidant enzymes (such as superoxide dismutase (SOD)), catalase and glutathione-related enzymes (such as glutathione peroxidase and glutathione S-transferases)) [4]. GSH acts as a principal antioxidant in the body by virtue of its free radical scavenging activity as well as its involvement in glutathione-dependent enzyme-catalyzed reactions [5]. Glutathione exists in two redox forms, namely, reduced (GSH) and oxidized (GSSG) glutathione. The relative concentrations of GSH and GSSG, i.e., the ratio of GSH/GSSG, reflects the cellular/mitochondrial glutathione redox status in the body. If antioxidant systems are overwhelmed by an excessive production of ROS, a decrease in GSH/GSSG ratio occurs, which can result in the development of pathological changes in tissues and/or organs. For example, impaired mitochondrial respiration, ischemia/reperfusion injury and auto-oxidation of catecholamines are the most common causes of oxidant-induced injury in the heart [6], whereas alcohol consumption, xenobiotic metabolism, environmental pollutants and irradiation are risk factors for oxidant-induced injury in the liver [7]. More importantly, a rapid depletion of cellular GSH can trigger programmed cell death (“apoptosis”), a process which cannot be reversed by the subsequent restoration of glutathione redox status [8]. In this regard, the search for effective antioxidant interventions for the amelioration of oxidant-induced tissue injury is of great interest in the area of preventive health.

8th Day SOD-Like Supreme (SOD-Like Supreme) is an antioxidant-enriched fermentation product with free radical scavenging properties. The main components of SOD-Like Supreme are germ extract (an extract of fermented fresh coffee bean, soybean and rice bran), oligosaccharides, green tea extract and ascorbic acid. The chemical analysis of the germ extract has shown it to contain flavonols (such as (+)-epigallocatechin gallate, (−)-epigallocatechin, (−)-epicatechin and d-(+)-catechin), nucleic acids, amino acids, vitamins and minerals [9,10]. The free radical scavenging activity of SOD-Like Supreme may be beneficial for protecting against oxidant-induced tissue injury. In the present study, the free radical scavenging activity of SOD-Like Supreme was assessed using an oxygen radical absorbance capacity (ORAC) assay. The effects of incubation with SOD-Like Supreme on menadione-induced toxicity and the associated changes in cellular GSH/GSSG ratio were examined in cultured H9c2 cardiomyocytes and AML12 hepatocytes. To extend the results obtained from the in vitro cell-based studies, the in vivo effects of acute pre- and post-treatment with SOD-Like Supreme on isoproterenol (ISO)-induced myocardial injury and carbon tetrachloride (CCl_4_)-induced hepatotoxicity were investigated in mice.

## 2. Materials and Methods

### 2.1. Chemicals and Reagents

H9c2 cardiomyocytes and AML12 hepatocytes were purchased from ATTC (Rockville, MD, USA). Dulbecco’s Modified Eagle’s Medium, Dulbecco’s modified Eagle’s medium/F12 and fetal bovine serum (FBS) were obtained from Thermo Fisher Scientific (Waltham, MA, USA). Sorbitol, nicotinamide adenine dinucleotide, GSH, GSSG, 3-(4,5-dimethylthiazol-2-yl)-2,5-diphenyltetrazolium bromide (MTT), ethylenediaminetetraacetic acid (EDTA), Trolox, ISO and menadione were purchased from the Sigma Chemical Co. (St. Louis, MO, USA). Assay kits for creatine kinase (CK) and aspartate aminotransferases (AST) were purchased from Stanbio Laboratory (Boerne, TX, USA). SOD-Like Supreme was supplied by Health Science Group Holdings Limited (Hong Kong SAR, China). The chemical composition of SOD-Like Supreme has previously been reported [9,10] (Table 1). SOD-Like Supreme was packaged in plastic vials using an aseptic vacuum technique that guarantees its effectiveness for 1.5 years without the addition of preservatives. For each experiment, a fresh vial of SOD-Like Supreme was used to ensure optimal activity. All other chemicals were of analytical grade. 

### 2.2. ORAC Assay

The in vitro antioxidant capacity of SOD-Like Supreme was determined by the ORAC assay. Briefly, an aliquot of 40 μL of SOD-Like Supreme, Trolox or ascorbic acid (at final concentrations of 0.0002, 0.001, 0.002 mg/mL in 50% v/v methanol in 75 mM potassium phosphate buffer, pH 7.4) was added to 120 μL of fluorescein solution (0.167 μM in potassium phosphate buffer), and the reaction mixture was incubated at 37 °C for 5 min. A 40 μL aliquot of 2,2′-azobis(2-amidinopropane) dihydrochloride (AAPH, 80 mM in potassium phosphate buffer) was rapidly added to the reaction mixture and the fluorescence intensity (with excitation and emission wavelengths of 485 and 520 nm, respectively) was recorded every minute for 80 min using a Victor V^3^ Multi-label Counter (Perkin-Elmer, Wellesley, MA, USA). A blank containing the vehicle (i.e., 50% v/v methanol in 75 mM potassium phosphate buffer) was also measured. A fluorescence decay curve was constructed by plotting the % initial fluorescence intensity versus time, and the area under curve (AUC) was determined. An ORAC curve of the sample was then obtained by plotting the value of AUC_sample_ − AUC_blank_ against the concentration of the sample. The ORAC value was estimated by comparing the slope of the ORAC curve of SOD-Like Supreme or ascorbic acid to that of Trolox and expressed as Trolox equivalents.

### 2.3. Cell Culture

H9c2 cells were cultured as monolayers in Dulbecco’s modified Eagle’s medium supplemented with 10% (v/v) FBS, 100 units/mL penicillin and 0.1 mg/mL streptomycin. AML12 cells were cultured as monolayers in Dulbecco’s modified Eagle’s medium/F12 supplemented with 10% (v/v) FBS, 100 units/mL penicillin and 0.1 mg/mL streptomycin. H9c2 cells and AML12 cells were kept at 37 °C in a humidified atmosphere of air containing 5% CO_2_. Cells used in the experiments were seeded at a density of 5 × 10^4^ cells or 2.5 × 10^4^ cells/well for H9c2 and AML12cells, respectively, on a 24-well culture plate, and cells in each well were grown to achieve 60–80% confluence within 24 h prior to drug incubation.

### 2.4. SOD-Like Supreme Incubation and Menadione Cytotoxicity

H9c2 cells or AML12 cells were co-incubated with SOD-Like Supreme (0.5, 1, 2 or 3% (v/v) in culture medium) and menadione (25 µM, 0.2% ethanol (v/v) in culture medium) with 1% FBS for 30 min at 37 °C. The non-menadione control group was incubated with vehicle (ethanol) only. Following incubation with menadione, cell viability and cellular GSH/GSSG ratios were measured by MTT assay and the enzymatic method of Griffith [11], respectively, in unchallenged and challenged cells, with or without SOD-Like Supreme incubation.

### 2.5. Animal Care

Adult Imprinting Control Region (ICR) female mice (8–10 weeks old, 25–35 g) were maintained under a 12 h dark/light cycle at approximately 22 °C, and allowed food and water ad libitum in the Animal and Plant Care Facility at the Hong Kong University of Science and Technology (HKUST). All experimental protocols were approved by the University Committee on Research Practice at the HKUST on 14 November 2016 (reference number: 2016086).

### 2.6. SOD-Like Supreme Treatment and Isoproterenol (ISO)-Induced Myocardial Injury

Adult female ICR mice were randomly divided into six groups of 9–10 animals each: (1) non-ISO controls; (2) non-ISO SOD-Like Supreme (0.69 mL/kg, which is the equivalent dose in humans according to the manufacturer’s specifications); (3) non-ISO SOD-Like Supreme (2.06 mL/kg); (4) ISO control; (5) ISO SOD-Like Supreme (0.69 mL/kg) and (6) ISO SOD-Like Supreme (2.06 mL/kg). Mice were orally administered SOD-Like Supreme by gavage a total of three times per day for two consecutive days (i.e., six doses in total). For the ISO-induced myocardial injury, mice were given ISO (150 mg/kg/day) intraperitoneally 30 min after the first SOD-Like Supreme treatment each day for two consecutive days, whereas the non-ISO mice received vehicle (i.e., saline) only. The second and third dosings with SOD-Like Supreme were done at 3 h and 6 h post-injection of ISO, respectively. Twenty-four hours after the final SOD-Like Supreme dosing, mice were anesthetized by intraperitoneal injection with a mixture of 100 mg/kg ketamine and 10 mg/kg xylazine in sterile saline. Heparinized blood samples were obtained from the ketamine/xylazine-anesthetized mice, and sacrificed by cardiac excision. 

### 2.7. SOD-Like Supreme Treatment and CCl_4_ Hepatotoxicity

Adult female ICR mice were randomly divided into six groups of 9–10 animals each: (1) non-CCl_4_ controls; (2) non-CCl_4_ SOD-Like Supreme (0.69 mL/kg); (3) non-CCl_4_ SOD-Like Supreme (2.06 mL/kg); (4) CCl_4_ control; (5) CCl_4_ SOD-Like Supreme (0.69 mL/kg) and (6) CCl_4_ SOD-Like Supreme (2.06 mL/kg). Mice were orally administered by gavage SOD-Like Supreme a total of three times on the day of the experiment. Thirty minutes after the first dosing with SOD-Like Supreme, mice were orally administered CCl_4_ at a dose of 1 mL/kg, while non-CCl_4_ animals received vehicle (i.e., olive oil) only. The second and third dosings with SOD-Like Supreme were done at 3 h and 6 h post-challenge with CCl_4_, respectively. Thirty minutes after the final dosing of SOD-Like Supreme, mice were anesthetized by intraperitoneal injection with a mixture of 100 mg/kg ketamine and 10 mg/kg xylazine in sterile saline. Heparinized blood samples were obtained from ketamine/xylazine-anesthetized mice, and animals were sacrificed by cardiac excision.

### 2.8. Preparation of Plasma and Mitochondrial Samples

Heparinized blood samples were centrifuged at 2150× *g* for 20 min at 4 °C. Samples of plasma supernatant were collected for biochemical analyses. Plasma CK and AST were measured using assay kits according to the manufacturer’s instructions. Sorbitol dehydrogenase (SDH) activities were measured as previously described [12].

Excised myocardial ventricular tissue samples were rinsed with ice-cold homogenizing buffer (50 mM Tris-HCl, 325 mM sucrose and 1 mM EDTA, pH 7.0). Liver tissue samples were rinsed with ice-cold homogenizing buffer (5 mM Tris-HCl, 250 mM sucrose and 0.1 mM EDTA, pH 7.0). Tissue homogenates were prepared by homogenizing ~0.5 g of minced tissue in 5 mL ice-cold homogenizing buffer (i.e., a 10% homogenate (w/v)) in a Teflon-in glass homogenizer (Glas-Col, Terre Haute, IN, USA) on ice at a speed of 40 rpm for 10 strokes. The homogenates were centrifuged (Himac CF 9RX, Hitachi Koki Co., Ltd., Ibaraki, Japan) at 600× *g* for 20 min at 4 °C. Mitochondrial fractions were obtained by centrifugation (Himac CR21G, Hitachi Koki Co., Ltd., Ibaraki, Japan) at 9200× *g* for 30 min at 4 °C.

### 2.9. Biochemical Analyses

After co-incubation with SOD-Like Supreme and menadione, H9c2 or AML12 cells were rinsed with phosphate-buffered saline (PBS) once followed by incubation with 300 μL MTT (1 mg/mL, in culture medium) for 3 h at 37 °C. Aliquots of 60 μL solubilization buffer (10% sodium dodecyl sulfate (w/v) and 45% dimethyl formamide (v/v), pH 4.7) were then added and the mixtures were incubated for 24 h at 37 °C. Cell viability was determined by measuring the absorbance at 600 nm (with a reference absorbance at 700 nm) using a Victor V^3^ Multi-label Counter. GSH and GSSG levels in cells or mitochondrial fractions were measured using the enzymatic method of Griffith [11]. Protein concentrations of mitochondrial fractions and cell lysates were determined using a BioRad protein assay kit (Hercules, CA, USA).

### 2.10. Statistical Analysis

Data shown in the Figures and Tables are the mean ± standard deviation (SD) or the mean ± standard error of the mean (SEM). Data were analyzed by one-way Analysis of Variance (ANOVA). Post-hoc multiple comparisons were performed using TUKEY or Dunnett T3 tests, depending on the results of homogeneity of variance test (Levene’s test); *p* values <0.05 were regarded as statistically significant. All statistical analyses were performed using GraphPad Prism 6.0 and SPSS 17.0.

## 3. Results

### 3.1. SOD-Like Supreme Displays Oxygen Radical Scavenging Activity In Vitro

SOD-Like Supreme demonstrated free radical scavenging activity, with a potency similar to that of Trolox (Table 2). However, the free radical scavenging activity of SOD-Like Supreme was found to be 1-fold more potent than that of ascorbic acid, as assessed by the slope of the ORAC curve.

### 3.2. SOD-Like Supreme Protects against Menadione Toxicity in Cultured Cardiomyocytes and Hepatocytes

**Figure 1:**
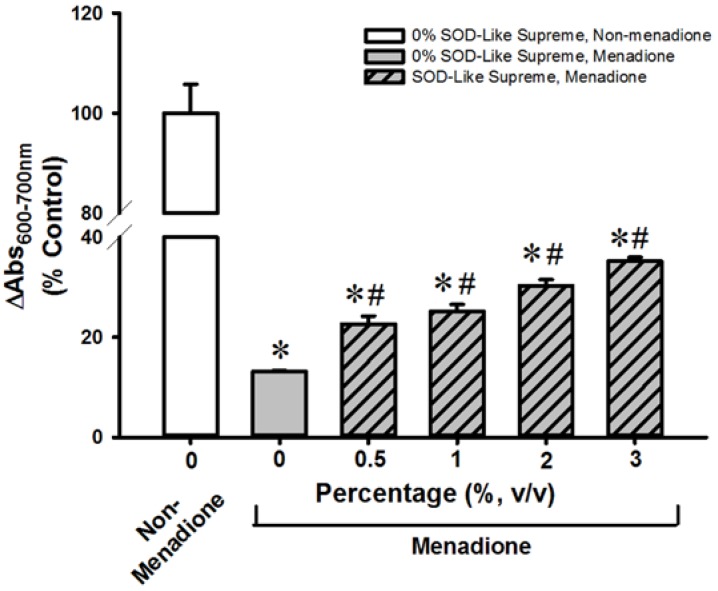
Effects of SOD-Like Supreme on cell viability of H9c2 cells following menadione challenge. After co-incubation of H9c2 cells with SOD-Like Supreme and menadione, cell viability was assessed by the MTT assay, as described in Materials and Methods. The difference between absorbance at 600 nm and absorbance at 700 nm of non-menadione control is 0.72 ± 0.04 (mean ± SD). Data are expressed as % of the non-menadione controls by normalizing relative to the value of non-menadione-incubated cells. Values given are means ± SD, with *n* = 4. * Significantly different from the non-menadione controls; # Significantly different from the menadione control group. SOD, superoxide dismutase; SD, standard deviation.

**Figure 2:**
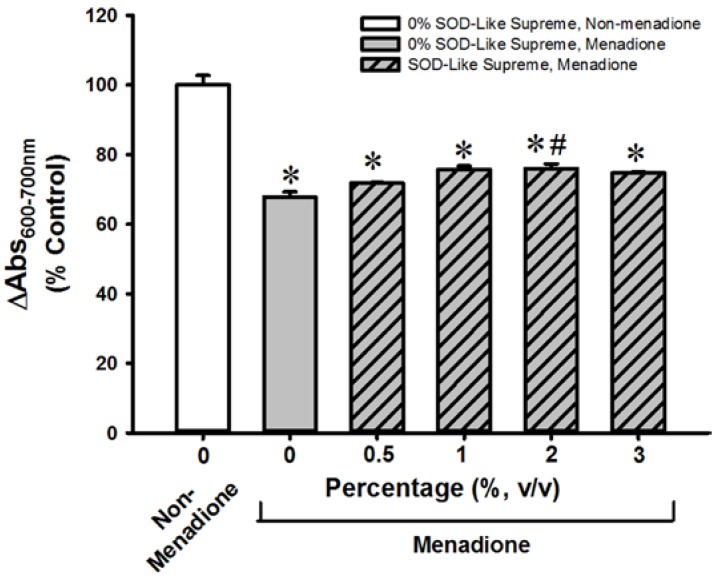
Effects of SOD-Like Supreme on cell viability of AML12 cells following menadione challenge. After the co-incubation of AML12 cells with SOD-Like Supreme and menadione, cell viability was assessed by the MTT assay, as described in Materials and Methods. The difference between absorbance at 600 nm and absorbance at 700 nm of non-menadione controls is 1.10 ± 0.03 (mean ± standard error of the mean (SEM) Data are expressed as % of the non-menadione controls by normalizing relative to the value of the non-menadione-incubated cells. Values given are means ± SEM, with *n* = 8. * Significantly different from the non-menadione controls; # Significantly different from the menadione control group.

**Figure 3:**
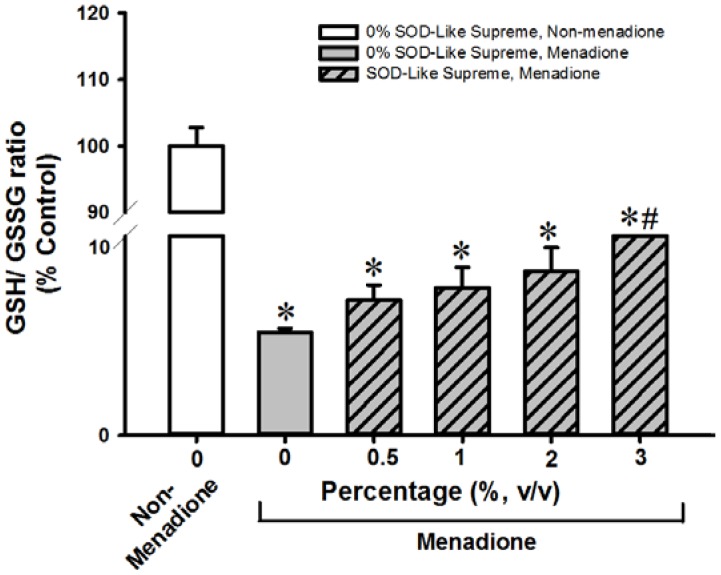
Effects of SOD-Like Supreme on cellular GSH/GSSG ratio in H9c2 cells following menadione challenge. After co-incubation of H9c2 cells with SOD-Like Supreme and menadione, cellular GSH/GSSG ratios were measured, as described in Materials and Methods. The GSH/GSSG ratio of the non-menadione controls is 38.4 ± 1.1 (mean ± SD). Data are expressed as % of the non-menadione controls by normalizing relative to the value of the non-menadione-incubated cells. Values given are means ± SD, with *n* = 4. * Significantly different from the non-menadione controls; # Significantly different from the menadione control group.

**Figure 4:**
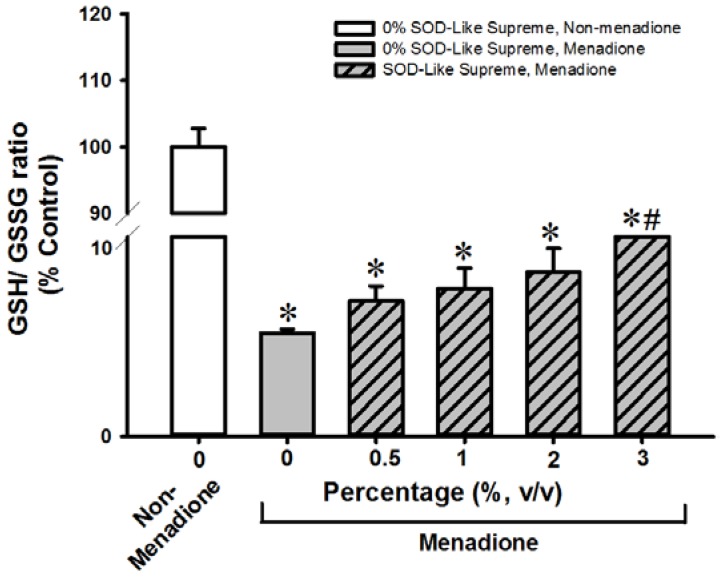
Effects of SOD-Like Supreme on cellular GSH/GSSG ratio in AML12 cells following menadione challenge. After the co-incubation of AML12 cells with SOD-Like Supreme and menadione, cellular GSH/GSSG ratios were measured, as described in Materials and Methods. The GSH/GSSG ratio of the non-menadione controls is 71.0 ± 2.0 (mean ± SD). Data are expressed as % of the non-menadione controls by normalizing relative to the value of the non-menadione-incubated cells. Values given are means ± SD, with *n* = 4. * Significantly different from the non-menadione controls; # Significantly different from menadione control group.

SOD-Like Supreme incubation did not affect cellular GSH/GSSG ratios or cell viability in non-menadione-challenged H9c2 cells or AML12 cells (data not shown). Incubation with menadione caused damage in H9c2 and AML12 cells, as indicated by decreases in cell viability (87% and 32%, respectively), when compared with the non-SOD-Like Supreme pre-incubated and menadione-unchallenged controls (Figure 1 and Figure 2. The loss of cell viability was paralleled by decreases in cellular GSH/GSSG ratios (97% and 94%) (Figure 3 and Figure 4). Pre-incubation with SOD-Like Supreme protected against menadione toxicity in H9c2 cells, as evidenced by an increase in cell viability, the degree of protection being 10–25% (Figure 1). The cytoprotection was associated with an increased GSH/GSSG ratio (31–105%, vs. menadione-challenged control) (Figure 3). Pre-incubation with SOD-Like Supreme protected AML12 cells against menadione toxicity, with the degree of protection being 12–25% (Figure 2). The cytoprotection against menadione in AML12 cells was paralleled by an increase in the cellular GSH/GSSG ratio (65–107%, vs. menadione-challenged control) (Figure 4).

### 3.3. SOD-Like Supreme Protects against ISO-Induced Myocardial Injury in Mice

**Figure 5:**
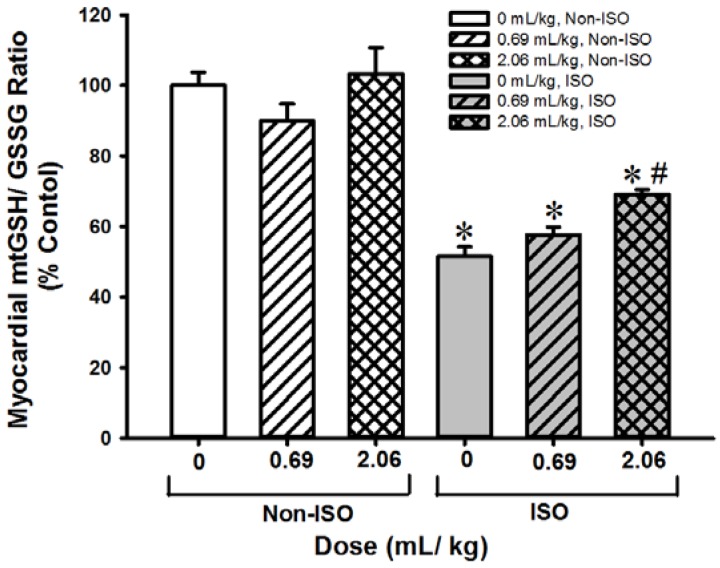
Effects of SOD-Like Supreme treatment on myocardial mitochondrial GSH/GSSG ratio in ISO-challenged mice. After treatment of ISO-challenged mice with SOD-Like Supreme, myocardial mitochondrial GSH/GSSG ratios were measured, as described in Materials and Methods. The GSH/GSSG ratio of the non-ISO control group is 14.7 ± 0.6 (mean ± SEM). Data are expressed as % of the non-ISO controls by normalizing relative to the value of the non-ISO control group. Values given are means ± SEM, with *n* ≥ 9. * Significantly different from the non-ISO controls; # Significantly different from the ISO control group.

Pre- and post-treatment with SOD-Like Supreme did not produce any detectable changes in mitochondrial GSH/GSSG ratios in heart tissues of non-ISO challenged mice. Injection of ISO-induced myocardial injury, as indicated by increases in plasma CK (90%, Table 3) and AST (37%, Table 4) activities as well as a decrease in the mitochondrial GSH/GSSG ratio (48%, Figure 5) in heart tissues. Pre- and post-treatment with SOD-Like Supreme (at 0.69 and 2.06 mL/kg) protected against ISO-induced myocardial injury, as evidenced by the reduction in plasma CK activity (17% and 51%). Treatment with a high dose of SOD-Like Supreme also reduced the plasma activity of AST (56%) in ISO-challenged mice. The cardioprotection afforded by SOD-Like Supreme pre-/post-treatment at the high dose was associated with an increase in the mitochondrial GSH/GSSG ratio (34%) in heart tissues of ISO-challenged mice.

### 3.4. SOD-Like Supreme Protects against CCl4 Hepatotoxicity in Mice

**Figure 6:**
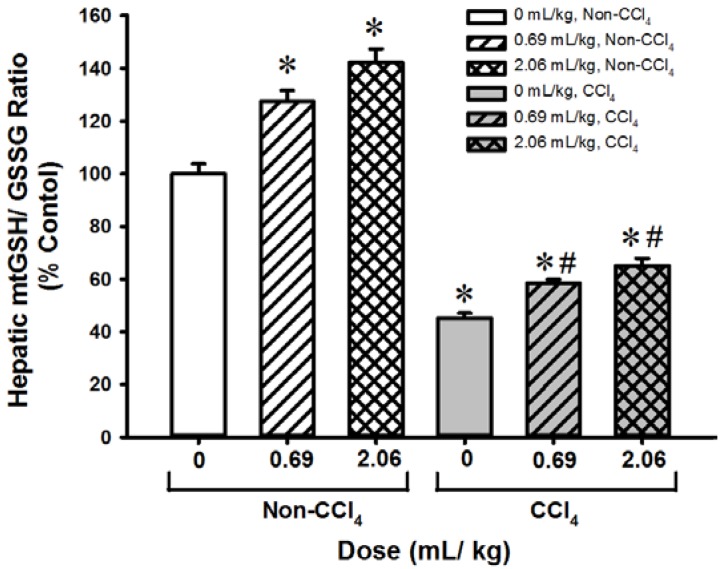
Effects of SOD-Like Supreme treatment on hepatic mitochondrial GSH/GSSG ratio in CCl_4_-challenged mice. After the SOD-Like Supreme treatment in CCl_4_-challenged mice, hepatic mitochondrial GSH/GSSG ratios were measured as described in Materials and Methods. The GSH/GSSG ratio of the non-CCl_4_ control group is 14.7 ± 0.6 (mean ± SEM). Data are expressed as % of the non- CCl_4_ control by normalizing relative to the value of the non- CCl_4_ control group. Values given are means ± SEM, with *n* ≥ 9. * Significantly different from the non-CCl_4_ controls; # Significantly different from CCl_4_ control group.

Pre-/post-treatment with SOD-Like Supreme (at doses of 0.69 and 2.06 mL/kg) increased plasma SDH (42% and 83%, respectively, Table 5) and AST (60% and 61%, Table 6) activities as well as the mitochondrial GSH/GSSG ratio (28% and 42%, Figure 6) in hepatic tissues. CCl_4_ challenge produced liver damage, as evidenced by increases in plasma SDH (112%) and AST (110%) activities as well as a decrease in the mitochondrial GSH/GSSG ratio (55%) in hepatic tissues. Pre-/post-treatment with SOD-Like Supreme protected against CCl_4_ hepatotoxicity, the extent of protection being 40% and 54 % (as assessed by plasma SDH activities), and 40% and 58% (as assessed by plasma AST activities), when compared with the non-SOD-Like Supreme-treated and CCl_4_-challenged controls. The hepatoprotection afforded by SOD-Like Supreme treatment was associated with increases in mitochondrial GSH/GSSG ratio (29% and 44%) in liver tissues of CCl_4_-treated mice.

## 4. Discussion

In the present study, SOD-Like Supreme was found to possess strong free radical scavenging activity in vitro, which was comparable to that of Trolox, an analog of α-tocopherol. To further investigate whether SOD-Like Supreme can exert antioxidant effects in cell-based and animal models subjected to oxidative stress, its effects on oxidant-induced injury were examined in cultured cardiomyocytes and hepatocytes in vitro as well as oxidant-induced injury in the myocardium and liver in mice in vivo. Menadione, which is a redox cycling agent that can induce cell death via ROS-dependent activation of poly ADP ribose polymerase (PARP) [13], was used to induce cytotoxicity in cultured cells. Incubation with SOD-Like Supreme was found to protect against menadione toxicity in H9c2 cardiomyocytes and AML12 hepatocytes. These protective effects were associated with elevations in cellular GSH/GSSG ratios in menadione-treated cardiomyocytes and hepatocytes. These observations are consistent with the notion that the maintenance cellular GSH/GSSG ratio is a critical indicator/predictor of cell survival. As mentioned earlier, the acute depletion of cellular GSH can induce apoptosis, which cannot be rescued by the subsequent restoration of glutathione redox status [8]. Since incubation with SOD-Like Supreme did not produce any detectable change in cellular GSH/GSSG ratios in H9c2 or AML12 cells, the maintenance of cellular glutathione redox status under conditions of oxidative stress is likely due to the sparing effect of SOD-Like Supreme on cellular GSH, presumably by virtue of its free radical scavenging activity. 

The cytoprotection afforded by SOD-Like Supreme in cultured cells was paralleled by results in in vivo models of oxidant-induced tissue injury. Acute pre-/post-treatment with SOD-Like Supreme was found to protect against ISO-induced myocardial injury and CCl_4_ hepatotoxicity in mice, as assessed by plasma CK, AST and SDH activities. Interestingly, pre-/post-treatment with multiple doses of SOD-Like Supreme also increased plasma SDH and AST activities 30 min after the final dosing. However, the activities of SDH and AST returned to control values at 24 h post-dosing with SOD-Like Supreme. In this connection, an earlier study has shown that organic alcohols (such as octanol and hexanol) can directly increase the activity of SDH [14]. Whether or not SOD-Like Supreme contains organic alcohols is yet to be determined. Given that the germ extract (one of the components of SOD-Like Supreme) contains various amino acids [9,10], the transient increase in plasma AST activity may be attributed to an increase in the concentration of substrates for AST in the blood immediately following the oral administration of SOD-Like Supreme. Consistent with the results obtained from the cell-based studies, the cardio/hepatoprotection against oxidant-induced injury was accompanied by increases in GSH/GSSG ratio in heart and liver tissues of SOD-Like Supreme-treated mice subjected to oxidative challenge.

ROS are causally involved in the development of ISO-induced myocardial injury [15] and CCl_4_ hepatotoxicity [16] and the removal of ROS by the scavenging action of SOD-Like Supreme would therefore be expected to protect against tissue injury produced by these agents. Preliminary studies have indicated that the in vivo tissue protection against oxidant injury by SOD-Like Supreme can only be achieved by acute and intermittent (but not long-term) administration. This observation suggests a relatively short in vivo half-life of the free radical scavenging activity of SOD-Like Supreme following its oral administration. Accordingly, a protocol of acute pre-/post-treatment with SOD-Like Supreme (i.e., one dose prior to oxidative challenge followed by three doses per day for two consecutive days or intermittent doses prior to sacrifice) was adopted in investigating the effect of SOD-Like Supreme on ISO-induced myocardial injury and CCl_4_ hepatotoxicity. The finding of cardioprotection and hepatoprotection afforded by SOD-Like Supreme pre-/post-treatment suggests that it would be necessary to administer SOD-Like Supreme in intermittent daily doses in order to achieve optimal free radical scavenging in humans. In this regard, catechins, which are present in SOD-Like Supreme, have been shown to protect against hepatotoxicity [17,18] and myocardial injury [19,20] in rodents. Given the low stability of catechins, Krupkova et al. [21] suggested that the preservation of catechin antioxidant activity would require its co-administration with other antioxidants, as is the case for SOD-Like Supreme.

## 5. Conclusions

In conclusion, SOD-Like Supreme incubation/treatment was found to protect against oxidant-induced injury in vitro (in cultured cardiomyocytes and hepatocytes) and in vivo (mouse heart and liver tissues), presumably by virtue of its free radical scavenging activity. Given the transient nature of its free radical scavenging properties, an intermittent oral administration of SOD-Like Supreme would seem to be the most effective way to confer an increase in in vivo antioxidant capacity when used in humans.

## Figures and Tables

**Table 1:** Chemical composition of SOD-Like Supreme.

Chemical Constituents	Percentage
Germ extract (an extract of fermented fresh coffee beans, soybeans and rice bran)	60%
Oligosaccharides	14%
DL-Alanine	2.5%
Green tea extract	1.2%
Dietary fiber (Pineapple fiber)	1.0%
Citric acid	0.6%
Vitamin C	0.5%

Chemical analysis has revealed that the germ extract contains flavonols (such as (+)-epigallocatechin gallate, (−)-epigallocatechin, (−)-epicatechin and d-(+)-catechin), nucleic acids, amino acids, vitamins and minerals [9]. The results of the chemical analysis are based on earlier studies [9,10] as well as on those obtained from the Japan Food Analysis Center.

**Table 2:** Oxygen radical scavenging activity of SOD-Like Supreme in vitro.

Antioxidant	Trolox Equivalents
	(mean ± SD)
Trolox	1.00 ± 0.12
Ascorbic acid	0.48 ± 0.12
SOD-Like Supreme	1.02 ± 0.06

The in vitro antioxidant capacity of SOD-Like Supreme was determined by the ORAC assay, as described in the Materials and Methods. Final concentrations of Trolox/ascorbic acid/SOD-Like Supreme used were 0, 0.0002, 0.001 or 0.002 mg/mL. The slope of the Trolox standard curve is 2259 ± 275 (mean ± SD). Data are expressed as Trolox equivalents by normalization relative to the value of the Trolox control group. Values given are means ± SD, with *n* = 5–6.

**Table 3:** Effects of SOD-Like Supreme treatment on plasma CK activity in mice with/without ISO challenge.

Group		% Control
		(mean ± SEM)
Non-ISO	Control	100 ± 5.44
	0.69 mL/kg	109 ± 3.15
	2.06 mL/kg	109 ± 6.30
ISO	Control	190 ± 6.67 *
	0.69 mL/kg	184 ± 6.96 *
	2.06 mL/kg	153 ± 8.65 *#

The plasma CK activity in the non-ISO control group is 29.0 ± 1.91 (average ± SEM). Data are expressed as % of the non-ISO controls by normalizing relative to the value of the non-ISO control group. * Significantly different from the non-ISO control group (*p* < 0.05, *n* ≥ 9); # Significantly different from the ISO control group (*p* < 0.05, *n* ≥ 9). CK, creatine kinase; ISO, isoproterenol.

**Table 4:** Effects of SOD-Like Supreme treatment on plasma AST activity in mice with/without ISO challenge.

Group		% Control
		(mean ± SEM)
Non-ISO	Control	100 ± 3.33
	0.69 mL/kg	101 ± 4.97
	2.06 mL/ kg	94.3 ± 3.47
ISO	Control	136 ± 2.08 *
	0.69 mL/kg	143 ± 5.33 *
	2.06 mL/kg	110 ± 4.35 #

The plasma AST activity of the non-ISO control group is 28.5 ± 1.31 (mean ± SEM). Data are expressed as % of the non-ISO controls by normalizing relative to the value of the non-ISO control group. * Significantly different from the non-ISO control group (*p* < 0.05, *n* ≥ 9); # Significantly different from the ISO control group (*p* < 0.05, *n* ≥ 9). AST, aspartate aminotransferases.

**Table 5:** Effects of SOD-Like Supreme treatment on plasma SDH activity in mice with/without CCl_4_ challenge.

Group		% Control
		(mean ± SEM)
Non-CCl_4_	Control	100.00 ± 3.75
	0.69 mL/kg	141.96 ± 6.75 *
	2.06 mL/kg	182.66 ± 13.23 *
CCl_4_	Control	271.75 ± 20.53 *
	0.69 mL/kg	209.32 ± 4.43 *
	2.06 mL/kg	234.25 ± 20.80 *

The plasma SDH activity of the non-CCl_4_ control group is 18.0 ± 0.69 (mean ± SEM). Data are expressed as % of the non-CCl_4_ controls by normalizing relative to the value of the non-CCl_4_ control group. * Significantly different from the non-CCl_4_ control group (*p* < 0.05, *n* ≥ 10). SDH, Sorbitol dehydrogenase.

**Table 6:** Effects of SOD-Like Supreme on plasma AST activity in mice with/without CCl_4_ challenge.

Group		% Control
		(mean ± SEM)
Non-CCl_4_	Control	100.00 ± 4.39
	0.69 mL/kg	160.20 ± 11.82 *
	2.06 mL/kg	160.53 ± 10.22 *
CCl_4_	Control	210.29 ± 12.41 *
	0.69 mL/kg	226.33 ± 16.31 *
	2.06 mL/kg	207.33 ± 8.09 *

The plasma AST activity of the non-CCl_4_ control group is 32.6 ± 1.43 (mean ± SEM). Data are expressed as % of the non-CCl_4_ controls by normalizing relative to the value of the non-CCl4 control group. * Significantly different from the non-CCl_4_ control group (*p* < 0.05, *n* ≥ 10).
